# An Unusual Case of Huge Tophaceous Pseudogout Mimicking as a Tumor-Like Lesion around the Ankle Joint: A Case Report and Literature Review

**DOI:** 10.1155/2019/9617184

**Published:** 2019-02-25

**Authors:** Mohammed Sadiq, Mayur Nayak, Ayesha Farheen, Vijay Digge

**Affiliations:** ^1^ESIC Medical College, Department of Orthopaedics, Kalaburgi, Karnataka, India; ^2^All India Institute of Medical Sciences (AIIMS), Department of Orthopaedics, Jay Prakash Narayan Apex Trauma Center, 5th Floor, Teaching Block, AIIMS Campus, New Delhi 110029, India; ^3^ESIC Medical College, Department of Pathology, Kalaburgi, Karnataka, India; ^4^All India Institute of Medical Sciences (AIIMS), Department of Orthopaedics, New Delhi, India

## Abstract

Pseudogout or calcium pyrophosphate dihydrate deposition disease (CPPD) primarily affects the joints and the periarticular tissues. Tophaceous or tumoral pseudogout is a rare form of this disease which is seen around the joints of extremities. It can be misdiagnosed as a neoplastic condition because of its clinicoradiological similarities, and thus, a proper histopathological examination is indispensable. We report one such case of extra-articular deposition of the CPPD crystals in a 65-year-old man who presented with an asymptomatic swelling around the left ankle. Radiographs showed a dense homogenous calcification, and FNAC revealed dense calcium deposits with numerous rhomboid-shaped crystals. It was managed by en bloc excision, and postoperative biopsy reports confirmed the diagnosis. Possibility of pseudogout should be kept as a differential diagnosis in patients presenting with calcified soft tissue swellings and should be subjected to a detailed histopathological examination for confirmation.

## 1. Background

Pseudogout or calcium pyrophosphate dihydrate deposition disease (CPPD) is characterized by accumulation of calcium pyrophosphate dihydrate crystals in the intra-articular and periarticular tissues [1]. Tophaceous pseudogout (tumoral CPPD) is a rare nonneoplastic form of the disease which primarily involves the temporomandibular joint, occasionally the perispinal tissues, and rarely the joints of extremities [2]. Owing to the similar clinicoradiological features, this condition can often be misdiagnosed as a neoplastic condition [1]. Thus, histopathology is the key to a correct diagnosis and appropriate management. This report documents an extremely rare case of a huge subcutaneous deposition of CPPD crystals in a patient presenting with a swelling on the lateral aspect of the left ankle joint.

## 2. Case Report

**Figure 1:**
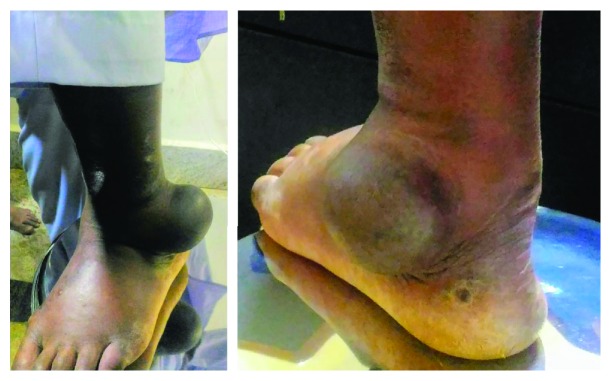
Swelling of 5 × 9 cm over the lateral aspect of the left ankle joint with discolored skin and no signs of inflammation.

**Figure 2:**
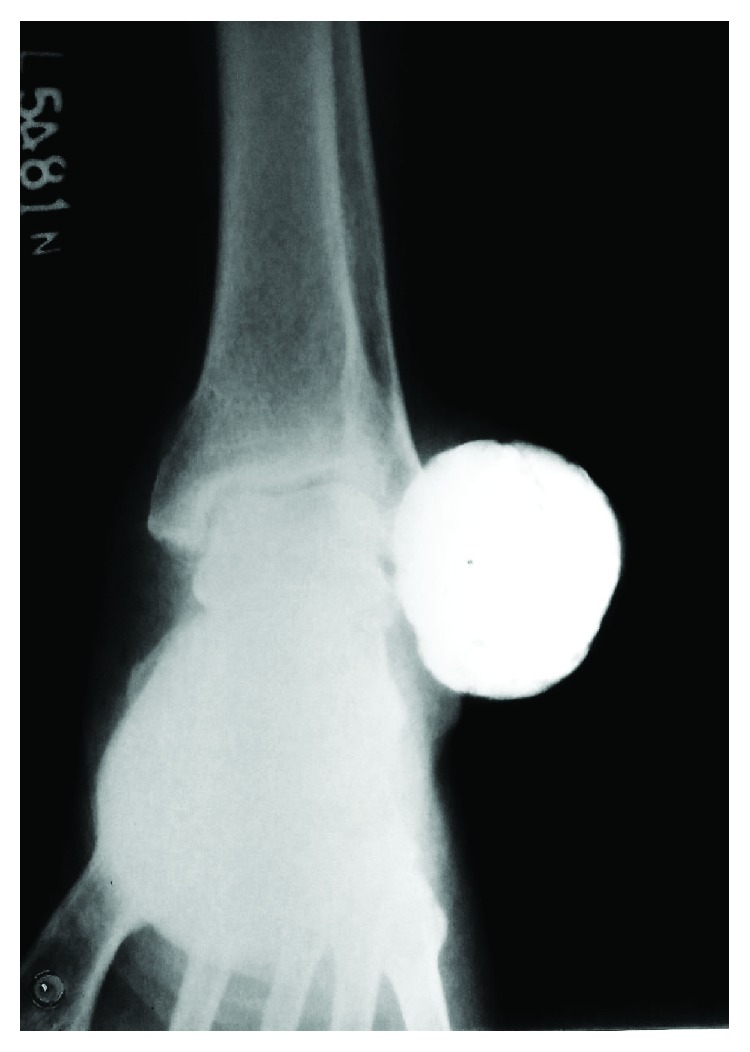
Anteroposterior X-ray of the left ankle joint showing a radiopaque mass with dense homogenous calcification over the lateral aspect of the ankle joint.

A 65-year-old man presented to the orthopaedic outpatient department with the chief complaint of swelling over the lateral aspect of his left ankle for the past two years. There was no history of any preceding trauma. The swelling did not increase in size and was not associated with any pain. On clinical examination, there was a 5 × 9 cm swelling over the lateral aspect of the left ankle joint (Figure 1), which was well defined, nonmobile, firm to hard in consistency, and nontender. The overlying and adjoining skin was discolored but without any evidence of thickening, induration, or increase in local temperature. There was no limitation of joint movements. There were no similar swellings in other parts of the body. An anteroposterior radiograph of the left ankle revealed a well-defined radiopaque lesion over the lateral aspect of the ankle joint. The lesion had an unusual homogenously calcified matrix with no areas of lysis within the mass. The lesion was sessile and was seen overlying the lateral malleolus. There was no periosteal reaction. The ankle joint appeared normal, and there were no pressure erosions over the lower end of the fibula (Figure 2). Blood investigations revealed normal serum calcium (10 mg/dl), phosphate (4 mg/dl), and alkaline phosphatase levels (127 U/l). The differential diagnoses of synovial chondromatosis, tophaceous gout, calcified lipoma, and myositis ossificans were considered, keeping in mind the possibility of malignant tumors such as synovial sarcoma, osteosarcoma, and chondrosarcoma.

**Figure 3:**
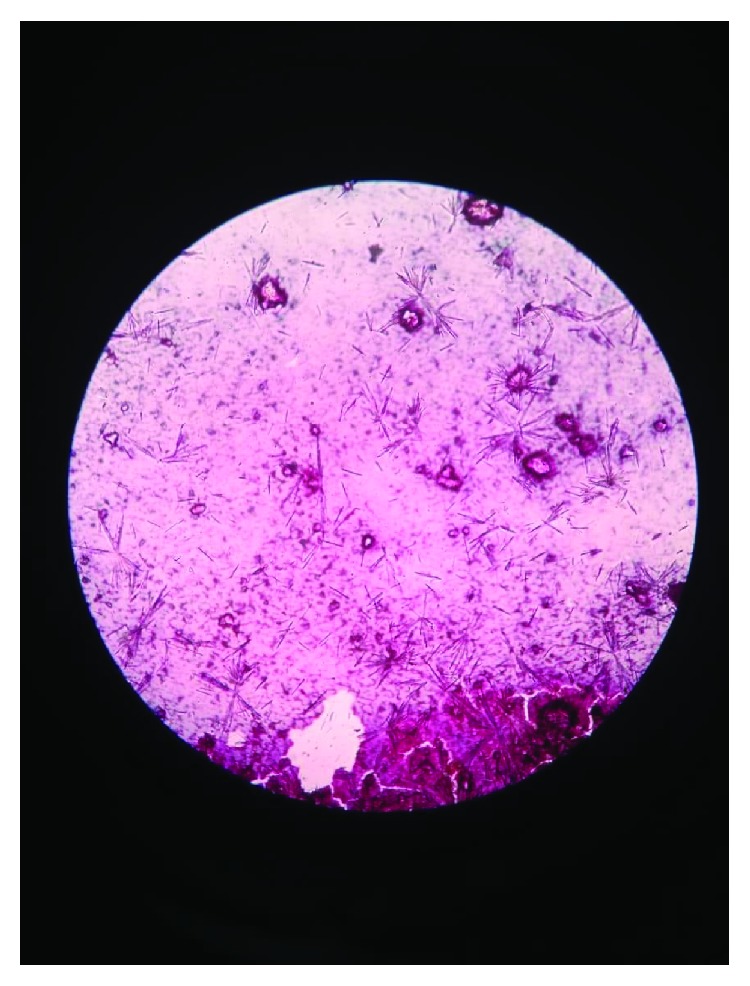
FNAC (Pap stain ×40) showing foci of calcium deposits with numerous highly refractile rhomboid-shaped crystals.

**Figure 4:**
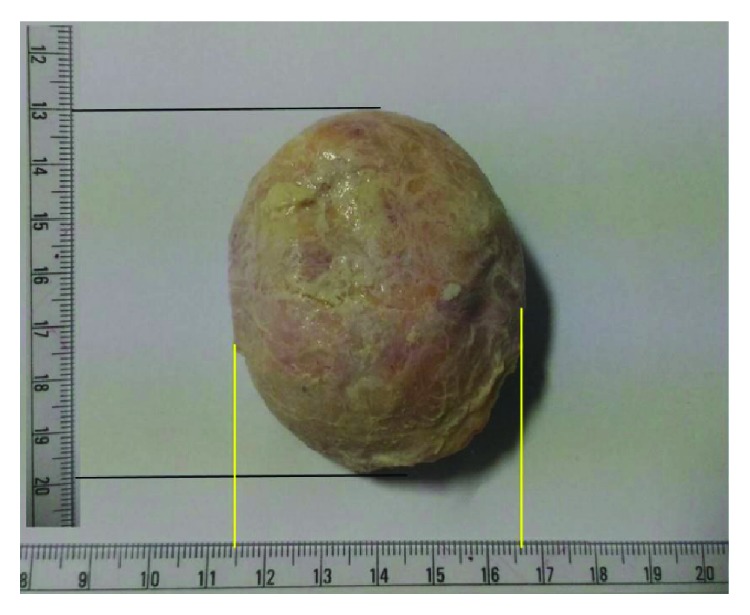
Ovoid-shaped mass measuring 7 × 5.5 × 4 cm reddish white in color after surgical removal.

**Figure 5:**
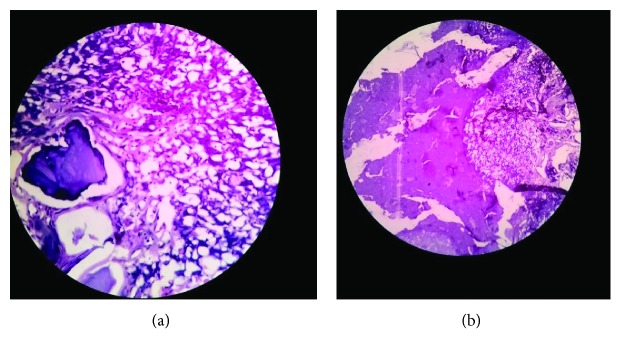
Biopsy: (a) (H&E stain ×200) large globular deposits of calcium with rhomboid-shaped crystals surrounded by mild inflammatory reaction; (b) (H&E stain ×400) CPPD crystals along with minimal lymphocytic infiltration and foci of calcification.

Considering the benign nature of the condition, a FNAC was done, and smears showed dense deposits of calcium with numerous refractile radiating rhomboid-shaped crystals which were seen against amorphous material (Figure 3). Based upon the FNAC report, a provisional diagnosis of calcium pyrophosphate deposition disease was made, and the patient planned for in toto excision of the mass. After obtaining a written informed consent, surgery was performed under spinal anesthesia. Intraoperatively, an ovoid-shaped mass, reddish white in color and measuring 7 × 5.5 × 4 cm, was noted underneath the peroneal tendon overlying the capsule (Figure 4). The mass was removed en bloc, and the wound was closed in layers. The postoperative course was uneventful, and the patient was discharged on the 4th day after wound inspection. The mass obtained was subjected to a histopathological examination, which confirmed the FNAC findings and showed large areas of calcium deposition with plenty of rhomboid-shaped refractile crystals (Figure 5). Hence, a final diagnosis of CPPD or tophaceous pseudogout was made. The patient is currently asymptomatic, and there has been no evidence of recurrence of the swelling till one-year postoperative follow-up.

## 3. Discussion

Calcium pyrophosphate dihydrate deposition disease (CPPD) is the most common form of crystal arthropathy second only to gout. It commonly presents as a monoarticular arthritis with crystal deposition in the synovial membranes, menisci, joint cartilages, and periarticular soft tissues. The knee is the most frequently involved joint followed by the wrist, shoulder, ankle, and elbow [3]. The risk factors include advanced age, metabolic disorders, osteoarthritis, previous joint injury, or any familial predisposition [3]. The diagnosis of the disease can be made by FNAC or a synovial fluid analysis. A polarizing light microscope shows weakly positive birefringent, rhomboid-shape crystals [3]. Tophaceous pseudogout is a rare condition characterized by deposition of CPPD crystals and is thought to arise due to chondrocyte metaplasia via intracellular proteoglycan that provides a seeding site for crystal formation [3]. It affects middle-aged or older individuals and has a female predominance [3, 4]. This condition commonly involves the temporomandibular joint, knee, and hand with a few reported cases of involvement of metacarpals and metatarsals; however, the ankle joint is rarely involved [1, 3–8].

It manifests as a soft tissue swelling forming a tumor-like mass due to deposition of the CPPD crystals [3] and can be seen in an intra- or extra-articular location. There have been a number of reports that describe the extra-articular presentation of this condition in certain anatomic locations such as the foot, proximal interphalangeal joint, and wrist [3, 5, 9] in addition to few other reports describing this condition to be intra-articular [2, 6, 10]. We found the mass to be extra-articular lying above the capsule underneath the peroneal tendons in the ankle joint. The size of the lesion reported has been variable. Our case presented as a huge swelling with a size as big as 7 cm in the longest dimension.

Typically, a case of pseudogout appears as a soft tissue mass of varying internal calcification often associated with erosion of adjacent bones [3, 9]. Sissons et al. reported two cases along with a literature review, wherein, he indicated that this condition presents as an enlarging periarticular mass with granular calcification along with evidence of bony destruction [11]. Kato et al. [10] reviewed CT findings of nine reported cases and found seven of them to have marginal calcification and two to have random calcification. Our case had an atypical presentation on imaging appearing as a large globular mass with homogenous calcification with no associated pressure signs in the adjacent bones, which further made it unusual.

A wide variety of conditions can simulate tophaceous pseudogout which include inflammatory conditions like tophaceous gout, myositis ossificans, benign conditions such as tumoral calcinosis, synovial chondromatosis, calcified lipoma, and BPOP, and malignant tumors such as synovial sarcoma, pleomorphic undifferentiated sarcoma, chondrosarcoma, and parosteal osteosarcoma [3, 12, 13]. The most important differentials are the inflammatory pathologies and the malignant diseases such as chondrosarcoma because they carry the highest risk of therapeutic misadventure. Tophaceous gout also originates in a periarticular location in hands and feet. However, it lacks any calcification as seen radiologically and exhibits needle-shaped monourate crystals in intra- and extracellular locations with the absence of calcification on histopathological examination [3]. Similarly, tumoral calcinosis also shares a similar location with tumoral CPPD, but it occurs predominantly in younger individuals and is more often multiple. Further, even with the similarity of the presence of hydroxyapatite deposits on histopathology, the definitive differentiating feature is an absence of a crystalline structure [5]. Chondrosarcoma is a malignant cartilaginous tumor and is seen in older population usually in the fourth to seventh decade and displays a variable calcific pattern [3], and there are previous reported cases of extraskeletal chondrosarcoma around the ankle joint [14]. Association of chondroid metaplasia which is frequently associated with tophaceous pseudogout makes the histological diagnosis difficult; however, the presence of giant cells and histiocytes with associated granulomatous lesions may help in differentiating the same from a chondroid tumor [4, 6, 15]. A few other conditions that form an important differential for this condition are listed in Table 1 along with their distinguishing features.

A case of tophaceous pseudogout involving the ankle joint has been previously reported by Seybold et al. [6]. However, the radiological image in this case showed nonspecific radiological calcification and cortical destruction of the talus whereas our patient presented with a diffuse homogenous calcification without any bony involvement. Secondly, they found the mass in the medial gutter of the ankle joint with an underlying talus and the deltoid ligament destruction whereas in our case, the mass was extracapsular and did not affect the underlying bones or ligaments. An interesting thing to note in this particular case is the initial misdiagnosis of a chondroma on both the preoperative CT-guided biopsy and postoperative histopathological examination, and it was only after one episode of recurrence that the final diagnosis of tophaceous pseudogout was made.

Therefore, to conclude, although a good histopathological study is indispensable for the diagnosis of this relatively rare disease in view of its clinicoradiological similarities with other diseases, the best approach would be a systematic one considering the clinical radiological and histological features in toto to obviate the need for repeated surgical interventions.

## 4. Conclusion

The present report is unique in itself in describing such a huge swelling of tumoral CPPD around the ankle joint without any bony destruction and an atypical radiological presentation. Demonstration of calcium phosphate dihydrate crystals in the calcified deposits helps us in differentiating it from benign or malignant calcifications. A holistic approach considering the clinical, radiological, and histological features is needed for appropriate management and outcomes.

## Figures and Tables

**Table 1:** Differential diagnosis for the patient in our case report and their distinguishing features.

	Site of origin	Most common distribution	Radiological appearance	Histological appearance
Tumoral pseudogout	Periarticular	Temporomandibular joint, perispinal tissues, joints of extremities	Radiopaque soft tissue mass with varying densities of calcification	Amorphous calcium deposits with numerous refractile radiating rhomboid crystals, demonstrates positive birefringence on polarized microscopy
Tophaceous gout	Periarticular	1st MTP joint, hands and feet	Juxtaarticular punched out erosions with sclerotic margins	Tophi—granulomatous inflammation with plenty of needle-shaped crystals. Negative birefringence on polarized microscopy
Tumoral calcinosis	Periarticular	Around large joints like the hip, shoulder, and elbow	Amorphous multilobulated (cloud-like) appearance	Lobules of calcific material surrounded by histiocytic giant cells
Synovial chondromatosis	Mainly intra-articular, sometimes in bursal tissues and tendon sheaths	Knee joint (70%), hip, shoulder, elbow	Calcified loose bodies in the joint, ring and arc appearances	Cartilage cells with varying degrees of atypia. Varying degrees of calcification and ossification
Myositis ossificans	Large muscle groups of the extremities	Around the knee, hip, and elbow	Circumscribed calcification with a lucent centre	Inner cellular zone, middle zone of woven bone, outer zone of mineralized bone
BPOP	Bony surfaces	Distal extremities, hands and feet	Continuous with the cortex with an underlying intact cortex	Chondro-osteoid matrix containing enlarged, bizarre, binucleated chondrocytes
Lipoma	Typically present in the subcutaneous plane	Usually found over the back, shoulders, and neck region but can be seen in other locations also	Calcifications are seen in 11% of the cases. Larger lipoma can result in bony erosions due to mass effect	Well-circumscribed encapsulated mass of mature adipocytes. Deeper lipomas may be associated with malignant features
Synovial sarcoma	Soft tissues of the body. In the extremities seen adjacent to large joints	Extremities, trunk, intrathoracic or intra-abdominal	Nonspecific calcification pattern	Biphasic or monophasic forms. Focal calcifications seen in 1/3rd of the cases
Chondrosarcoma	Long bones	Femur, pelvis	Ring and arc (popcorn) types of calcifications	Focal calcifications with no osteoid or bone formation
Parosteal osteosarcoma	Metaphysis of long bones	Distal femur, proximal humerus, proximal tibia	Large lobulated exophytic mass, usually circumferential involvement, central dense ossification; string sign—radiolucent line separating the tumor from the cortex	Low grade tumor. Well-formed bony trabeculae and osteoid deposition with or without osteoblastic rimming; stromal cells show mild cellular atypia and few mitoses
